# Supplementary material for: CT-Derived Body Composition Is a Predictor of Survival after Esophagectomy
Source: J Clin Med. 2023 Mar 8;12(6):2106. doi: 10.3390/jcm12062106 (PMC10058526; doi:10.3390/jcm12062106)

Supplemental Table S1. Summary of tumor and body composition feature information (n=183).

Characteristic	Value[1]
**Tumor volume (ml)**	58.73±(42.66)
**Tumor density (HU)**	23.21±(37.17)
**Tumor mean diameter (mm)**	17.35±(8.00)
**Tumor maximum length (mm)**	62.66±(44.26)
**VAT volume (ml)**	5.91±(3.21)
**VAT density (HU)**	-87.63±(7.36)
**VAT mass (kg)**	6.13±(3.31)
**SAT volume (ml)**	17.17±(9.63)
**SAT density (HU)**	-90.82±(10.08)
**SAT mass (kg)**	17.72±(9.85)
**IMAT volume (ml)**	1.38±(0.77)
**IMAT density (HU)**	-72.71±(5.75)
**IMAT mass (kg)**	1.46±(0.80)
**SM volume (ml)**	17.43±(4.49)
**SM density (HU)**	36.60±(8.58)
**SM mass (kg)**	20.51±(5.29)
**Bone volume (ml)**	4.56±(0.86)
**Bone density (HU)**	366.30±(43.31)
**Bone mass (kg)**	7.03±(1.39)
**1Mean±(SD)**	

Supplemental Table S2. Variables used in the SVM model to predict one-year post-esophagectomy survival.

**Variable**	**Category**
Race	Clinicodemographic
**BMI**	Clinicodemographic
Pathological n stage	Clinicodemographic
Effusion	Post-operative complication
**Pneumonia**	Post-operative complication
**DV**	Radiomic
**SUV P75**	PET
Bone density	Body composition
Bone mass	Body composition
VAT density	Body composition

Supplemental Table S3. Variables used in the random forest model to predict three-year post-esophagectomy survival.

Variable	Category
Mean.2	Radiomic
Percentile10.3	Radiomic
RootMeanSquared.3	Radiomic
SumAverage.2	Radiomic
SumAverage.4	Radiomic
GLCM: Autocorrelation.3	Radiomic
Minimum SUV	PET
Race	Demographic
Bone mass	Body Composition
IMAT mass	Body Composition
Pathological M stage	Demographic
Pathological T stage	Demographic
Smoking history	Demographic
IMAT volume	Body Composition

Supplemental Table S4. Variables used in the SVM model to predict five-year post-esophagectomy survival.

Variable	Category
Mean.4	Radiomic
Median.4	Radiomic
Percentile10.2	Radiomic
RootMeanSquared.3	Radiomic
Age	Demographic
BMI	Demographic
VAT mass	Body composition
Pathological T stage	Demographic
Tumor mean diameter	Tumor
IMAT volume	Body composition

Supplemental Figure S1

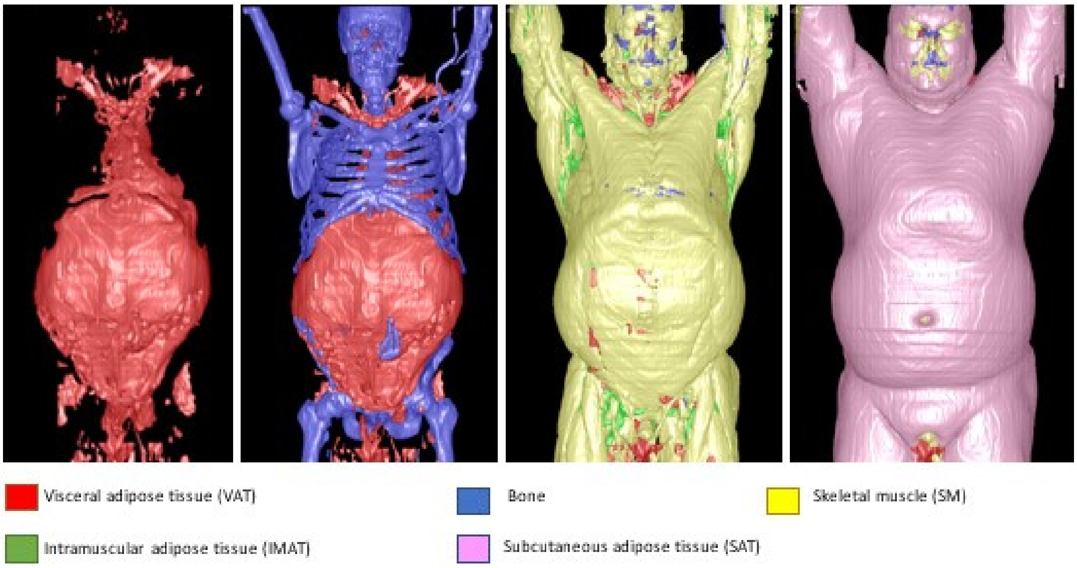

Supplemental Figure S2

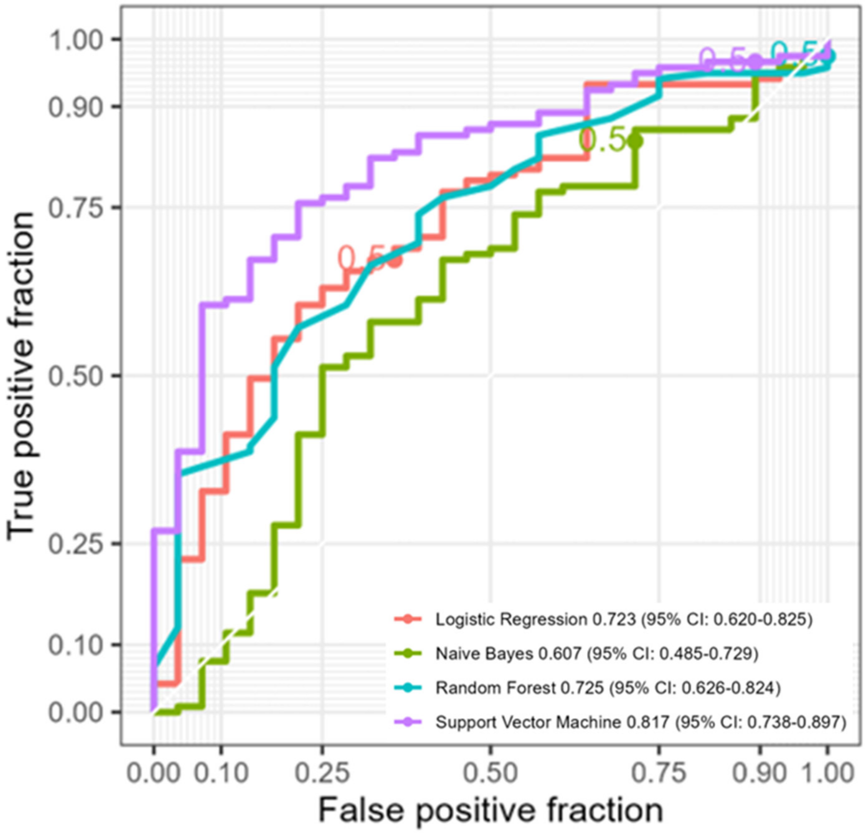

Supplemental Figure S3

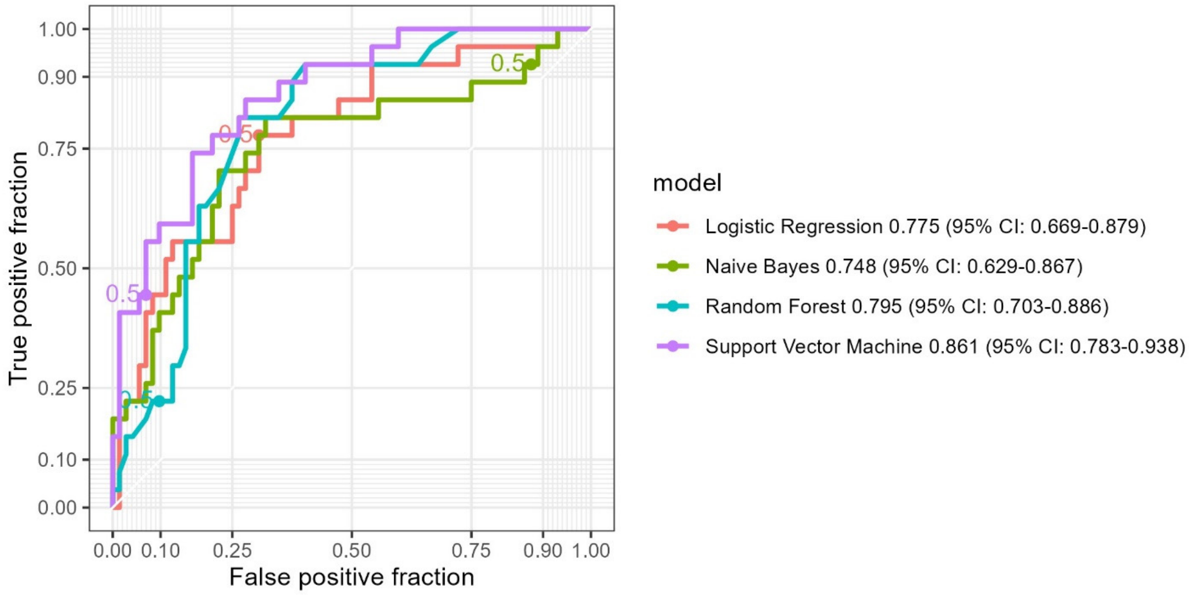

Supplemental Figure S4

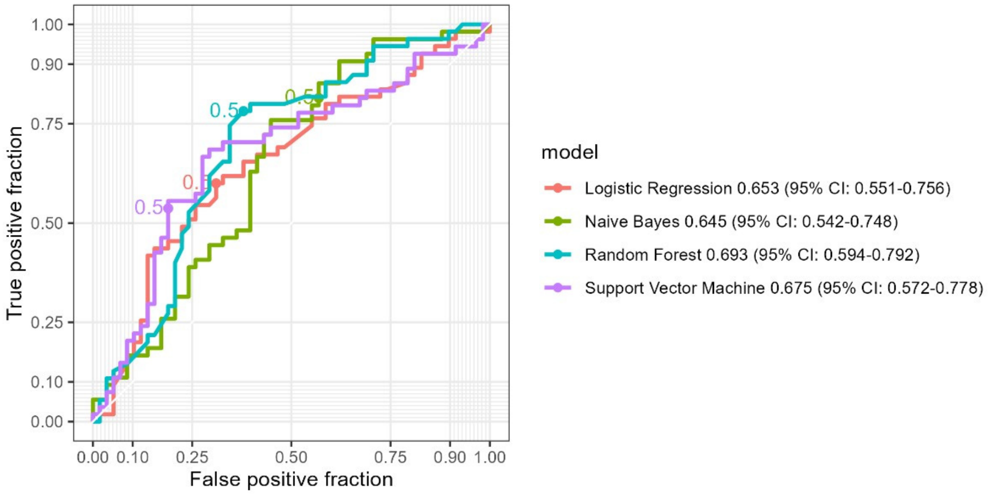

Supplement: Supplementary file 1 [file jcm-12-02106-s001.zip › jcm-2213768-supplementary.pdf]
